# Theoretical analyses of resonant frequency shift in anomalous dispersion enhanced resonant optical gyroscopes

**DOI:** 10.1038/srep38759

**Published:** 2016-12-12

**Authors:** Jian Lin, Jiaming Liu, Hao Zhang, Wenxiu Li, Lu Zhao, Junjie Jin, Anping Huang, Xiaofu Zhang, Zhisong Xiao

**Affiliations:** 1 Beihang University, School of Physics and Nuclear Energy Engineering, Key Laboratory of Micro-Nano Measurement-Manipulation and Physics (Ministry of Education), Beijing, 100191, China

## Abstract

Rigorous expressions of resonant frequency shift (RFS) in anomalous dispersion enhanced resonant optical gyroscopes (ADEROGs) are deduced without making approximation, which provides a precise theoretical guidance to achieve ultra-sensitive ADEROGs. A refractive index related modification factor 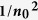 is introduced when considering special theory of relativity (STR). We demonstrate that the RFS will not be ”infinitely large” by using critical anomalous dispersion (CAD) and negative modification does not exist, which make the mechanism of anomalous dispersion enhancement clear and coherent. Although step change of RFS will happen when the anomalous dispersion condition varies, the amplification of RFS is limited by attainable variation of refractive index in practice. Moreover, it is shown that the properties of anomalous dispersion will influence not only the amplification of RFS, but also the detection range of ADEROGs.

Resonant optical gyroscopes (ROGs) operate on the principle of the Sagnac effect^1^, i.e. the rotation of the optical cavity will change its resonant frequency for the light propagating in it. The resonant frequency difference between clockwise (CW) and counterclockwise (CCW) directions can be measured accurately by optical heterodyne technique like in the cases of Ring Laser Gyroscopes (RLGs), which are active resonant optical gyroscopes. In recent years, there has been interest in the development of resonant optical gyroscopes with novel materials^2^,^3^,^4^, structures^5^,^6^,^7^,^8^,^9^,^10^,^11^,^12^ and physical effects^13^,^14^,^15^,^16^,^17^,^18^,^19^, among which the utilization of fast light for sensitivity enhancement is a potential technique^15^,^16^,^17^,^18^,^19^. Actually, it is the strongly anomalous dispersion that amplifies the Sagnac RFS as well as allows superluminal light propagation. There are two schemes to generate anomalous dispersion in ROGs, i.e. coupled-resonator structures^15^,^16^,^17^ and steep negative dispersion materials^18^,^19^.

In the superluminal helium–neon RLGs^15^,^16^,^17^, the amplification of RFS is achieved by replacing one mirror in the traditional ring laser cavity with auxiliary passive cavities, where a theoretical rotation sensitivity enhancement of about two orders of magnitude is demonstrated^15^. The performance of such gyros can be regulated by changing the mirrors’ properties in these coupled cavities^16^ or the round-trip length of the ring lasers and the coupled cavities^17^. However, the sensitivity of enhancement factor to rotation or equivalent frequency detuning requires real time control of complex reflection coefficient of the mirrors, which is hard to achieve in assembled devices. Moreover, the direct relation between RFS and coupled cavities induced anomalous dispersion is not revealed, because coupled condition is considered instead of dispersion condition in these works.

The optical resonator with intracavity dispersive cell is another scheme for ADEROGs^18^,^19^. Compared to structure induced dispersion, the dispersion in materials is well studied and a number of different media can carry out anomalous dispersion, such as semiconductors^20^, dye solutions^21^, room temperature solids^22^, optical fibres^23^ and atomic vapors^24^. The dispersive element can be treated as both an amplitude and phase nonlinear filter^18^. However, the time-dependent analysis of the cavity field is intuitionistic and hard to understand. A simpler approach can be used to analyze the influence of anomalous dispersion, where the effect of Fresnel drag is considered for preciseness^19^. According to their results, the dispersion-related modification factor of RFS is 1/*n*_*g*_, where *n*_*g*_ = *n*(*ω*) − *Kω* is the group index. Obviously, the RFS will be amplified with the group velocity be faster than the speed of light in vacuum if *n*_*g*_ is less than 1. This is the reason that such amplification is connected with fast light^15^,^16^,^17^,^18^,^19^. Nevertheless, the modification factor is not accurate as some approximations are made in their derivations, which lead to some inappropriate conclusions. For examples, it is demonstrated that RFS will be “infinitely large” by using CAD (corresponding to the group index approaching zero). Besides, confusing negative modification for RFS is possible when the group index is less than zero.

In this paper, we theoretical analyze the RFS in ADEROGs based on concise optical resonator theory. Results of non-dispersion case and anomalous dispersion case are presented and compared. To provide a strict derivation of the expression for the magnitude of the Sagnac effect in the framework of STR, Lorentz velocity transformation is used and no approximation is made in our derivations. The amplification factor of RFS is hence not the inverse of group index, which lead to some distinct conclusions. The influences of anomalous dispersion and relativistic velocities are presented by numerical calculation and detailed discussed.

## Results

**Figure 1:**
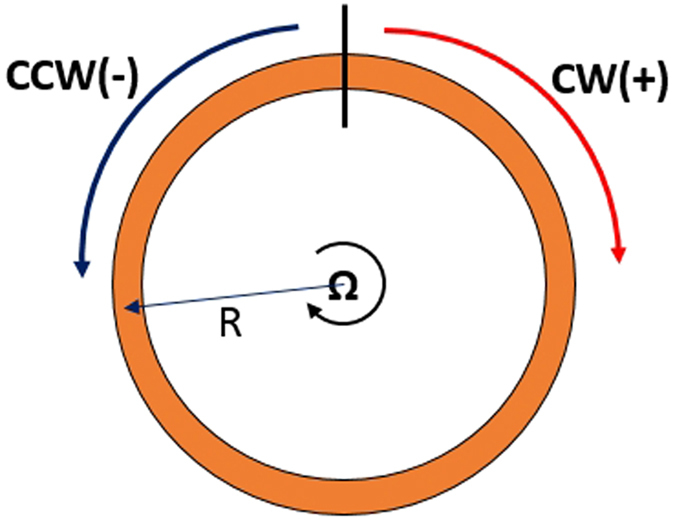
Diagram of the single ring resonator with radius *R*. The cavity is subject to rotation at an angular rate of Ω, and the medium inside the cavity is non-dispersive or anomalous dispersive material. Resonant light in CW and CCW directions counter-propagate in the cavity simultaneously.

According to Sagnac effect^1^, any closed curve with “non-zero vector area” can compose the optical path for detecting rotation. However, path-integral approach is needed in the analysis of polygon-shaped cavities. To focus on the physical effect and avoid complicated mathematics, a ring cavity with radius R is considered. As is shown in Fig. 1, the cavity is filled with non-dispersive material (for example, vacuum) or anomalous dispersive one for comparison. We assume that the cavity clockwise rotates at angular velocity Ω, i.e. the rotation velocity is *v* = Ω*R*. Results of counterclockwise rotation can be calculated in the same way used below. In addition, the CW and CCW light are presented by the superscript “+” and “−” respectively.

The optical resonator is the sensitive element of ROGs. When the light propagating in the resonator satisfies the resonant condition, the transmission will reach minimum. The resonant condition means completely coherence of the circulating light, which requires the optical length to be an integral multiple of its wavelength:


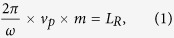


where *ω* is the angular frequency of the light, *v*_*p*_ is the equivalent phase velocity, *m* is arbitrary positive integer and *L*_*R*_ is the effective optical length which will be modified by rotation in gyros. Illustrated below are the expressions for the changed path length *L*_*R*_ in a laboratory (stationary) system of coordinates^25^:


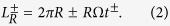


Here, 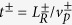 are the times spent by counterpropagating waves to complete one trip around the enclosed area of the ring, while 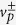 is the corresponding equivalent phase velocity.

Apparently, the resonant frequency will be changed by rotation too. The increase of *L*_*R*_ will lead to the decrease of *ω*, and vice versa. In other words, the resonant frequency *ω*_0_ at rest will become 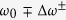 when the cavity rotates (Δ*ω* is the absolute value of RFS). The shift of the resonant frequency is the key factor that dispersion works, as it is proved that the Sagnac phase shift is independent of medium parameters when the frequencies of counter-propagating waves are same^25^. Things are different when frequency changes. Let us take the developing process of Δ*ω*^+^ as example. The rotation-induced increase of effective optical length requires the decrease of resonant frequency to make the equation (1) still true. Hence the phase velocity *v*_*p*_ = *c*/*n* (*c* refers to the speed of light in vacuum and *n* is the refractive index) decreases owning to the negative correlation between refractive index and frequency in anomalous dispersion medium, which will further cause the decrease of frequency. This process is iterative until Δ*ω*^+^ reaching a steady-state value, which is greater than the original one. Accurate expressions are demonstrated below.

The equivalent phase velocity and effective optical length of the resonant light in stationary cavity are *v*_*p*_ = *c*/*n*_0_ and L_R_ = 2*π*R respectively, where *n*_0_ is the corresponding refractive index. So we can get the resonant frequencies of CW and CCW light:


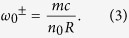


For rotation case, owing to the dispersion, the phase velocities should change into:


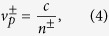


where *n*^±^ are the refractive index for 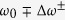. According to equations (1), (2) and (4), Δ*ω*^±^ are given by (subscripts “1” corresponds to the non-relativistic RFS):


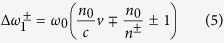


Actually, the STR works when two coordinate frames move at constant velocity relative to each other, which is just the case happening for light propagating in rotating cavity. The expressions for the Sagnac effect in the framework of STR can be derived by taking advantage of the invariance of the interval *x*^2^ + *y*^2^ + *z*^2^ = *c*^2^*t*^2^ (where *x*, *y*, *z* are the wave front coordinates, and *t* is the time)^26^. A simpler and physically more illustrative way is based on the relativistic law of velocity composition^25^:


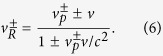


The general expressions of RFS in consideration of STR are deduced by combining equations (1), (2) and (6) (subscripts “2” corresponds to the relativistic RFS):


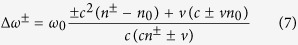


where *n*^±^ are nonanalytic above. We will introduce concrete expressions of *n*^±^ in different kinds of media next to get computable RFSs. For cavity with non-dispersive medium, *n*(*ω*^±^) = *n*_0_, the RFSs become (subscripts “n” represents non-dispersive case):


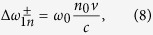



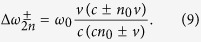


Obviously, the RFS is modified when the effect of STR is considered. The influence is rotation related just as the relativistic phase velocity does. Because 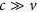, we can also get 
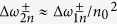
, which is in accord with the previous research^19^.

For cavity with anomalous dispersive medium, *n*(*ω*^±^) = *n*_0_ ± Δ*n*, where Δ*n*^±^ are the shifts of refractive index for frequency transfers from *ω*_0_ to 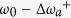 for CW light or 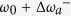 for CCW light. Normally, the shifts are regarded as linear variation^27^:


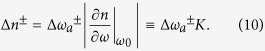


The width of such dispersion Δ*ω* and maximum dispersion gradient *K*_*max*_ are main token parameters in experiments, which lead to limited variation of the refractive index Δ*n*. For example, the *K*_*max*_ varies from 10^−14^ to 10^−9^ with Δ*n* up to 10^−7^~10^−5^ in typical anomalously dispersive atomic media^24^. The limited Δ*n* will confine the width of RFS and is an important condition for our following discussion, as some results will go against it, i.e. is unpractical. The potential performance of ADEROGs is therefore influenced.

Substituting equation (10) into equation (5) while keeping in mind that Δ*ω*^±^ are assumed to be positive, we can get the non-relativistic RFSs (subscripts “a” represents anomalous dispersive case):


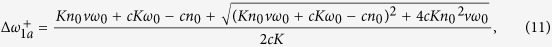



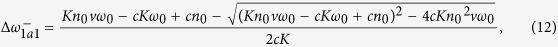



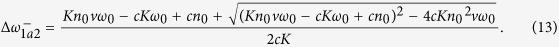


Note that there is an implied condition for CCW light, i.e. *Kn*_0_*vω*_0_ − *cKω*_0_ + *cn*_0_ > 0, which can be written as *K* < *cn*_0_/(*cω*_0_ − *ω*_0_*n*_0_*v*). As the RFSs of CW and CCW light should be detected simultaneously, the restriction on the range of K for CCW light is of essential importance in the determination of properties of anomalous dispersion. In addition, the existence of two positive solutions for CW light requires careful discussion. We will demonstrate later that 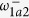 is unpractical and useless in detection.

Relativistic RFSs are obtained in similar way:


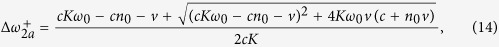



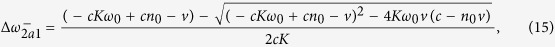



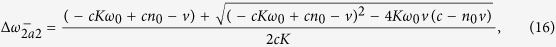


where −*cKω*_0_ + *cn*_0_ − *v* > 0 is the corresponding restriction.

The results above are somehow complicated, which involve all physical details. Obviously, the dispersion-related modification factor of RFS is no more the inverse of group refractive index, which eliminates the confusing infinite-enhancement and negative-modification of RFS. Detailed discussions will be presented in next section.

## Discussion

In this section, we will analyze the performance of ADEROGs by numerical simulation. While a number of different media can carry out steep negative dispersion^20^,^21^,^22^,^23^,^24^, atomic media offer a flexible platform for such gyros through the ability to readily control and vary the atomic parameters that influence the dispersion. We will hence choose ^85^*Rb* vapor as the anomalous dispersion materials inside our resonator here. In addition, it is vacuum that acts as the comparative non-dispersion material. Thus, the refractive index *n*_0_ for resonant frequency at rest is 1. The perimeter of the resonator is set as 78 cm to make the original resonant wavelength center on 780 nm, i.e. *ω*_0_ ≈ 2.4166 × 10^15^ Hz, which is the typical wavelength that experiences strongly anomalous dispersion in related research^24^,^28^. In fact, any perimeter that satisfy equation (1) is proper.

**Figure 2:**
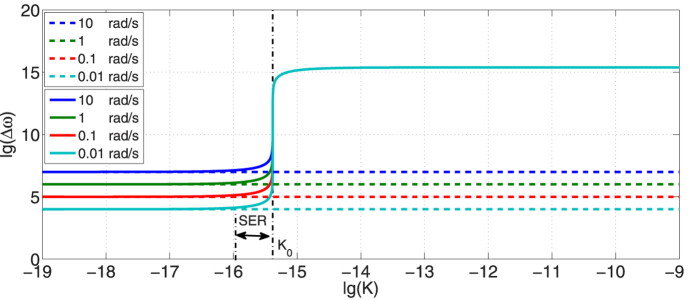
Results of 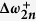 (dashed lines) and 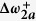 (solid curves) versus the dispersion gradient *K* in different angular velocities. The SER refers to strong enhancement region and *K*_0_ is the threshold value where step change happens in 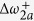. The range of K comes from the experiments^24^.

Figure 2 presents the RFSs of non-dispersion cases 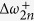 and anomalous dispersion cases 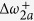 in different angular velocities, while the influence of STR is considered. According to equation (9), the RSF in non-dispersion condition is approximately equal to *ω*_0_Ω*R*/*c*, so 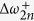 is independent with the dispersion gradient *K* and is parallel to horizontal axis. Because radius *R* is fixed, 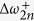 is proportional to the angular velocity Ω, which agrees with the previous researches^1^,^25^,^26^. Similarly, 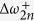 will be proportional to the radius *R* when angular velocity Ω is fixed, which provides a practical method to increase the sensitivity of optical gyroscopes by increasing their size. For profiles of 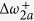, there is a threshold value for *K*. When *K* is much smaller than the critical *K*_0_, i.e. the influence of anomalous dispersion is indistinctive, 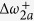 is almost equal to 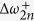. Evident amplification of RFS is achieved when *K* approaches *K*_0_ and we call this scope as strong enhancement region (SER). If *K* > *K*_0_, step transition of 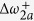 will happen, and 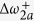 is amplified by great extent compared to 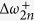.

**Figure 3:**
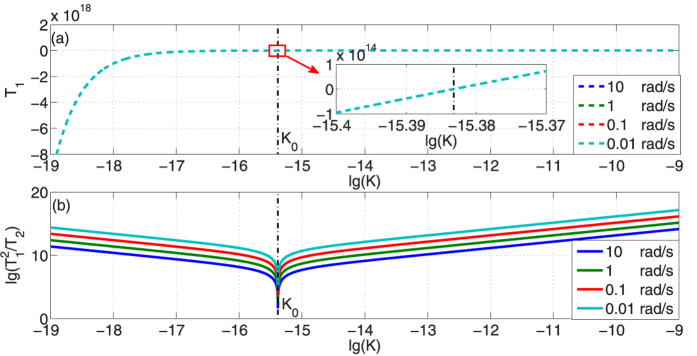
(**a**) *T*_1_ versus the dispersion gradient *K* in different rotation velocities. The profiles of different rotation velocities overlap while *K*_0_ refers to the transition of *T*_1_ (**b**) Value of 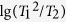 versus the dispersion gradient *K* in different rotation velocities. 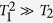 in most cases while 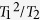 decreases rapidly on the scope near *K*_0_.

To explain this step change, we firstly rewrite the equation (14) as:


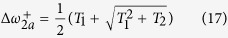


where 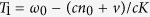, 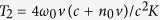. Because 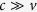, the value of *T*_1_ is hardly changed by rotation velocity *v*, while *T*_2_ is nearly proportional to *v*. As is shown in Fig. 3(a), there is a transition value for *T*_1_, which is exactly the critical *K*_0_ and its expression is 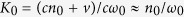. *T*_1_ is negative when *K* < *K*_0_ and positive when *K* > *K*_0_. And it can be seen from Fig. 3(b) that 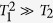 except on the scope near *K*_0_, which means that 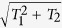 is slightly greater than |*T*_1_| in most cases. Thus, when *K* < *K*_0_, 
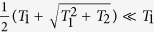
 and the value of *T*_2_ plays an important role in the final result, which eventually leads to the shift of 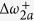 in different angular velocities. The influence of *T*_2_ increases as the ratio between 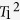 and *T*_2_ decreases, resulting in the existence of SER. As for the cases of *K* > *K*_0_, 
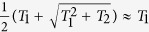
, where the influence of tiny *T*_2_ is inconspicuous. It is important to emphasize that the great amplification of RFS when *K* > *K*_0_ is useless for detection as it is nearly same in different rotation velocities. In fact, it is unrealizable in practice, because the change of refractive index Δ*n* is beyond the available value in experiments^24^.

**Figure 4:**
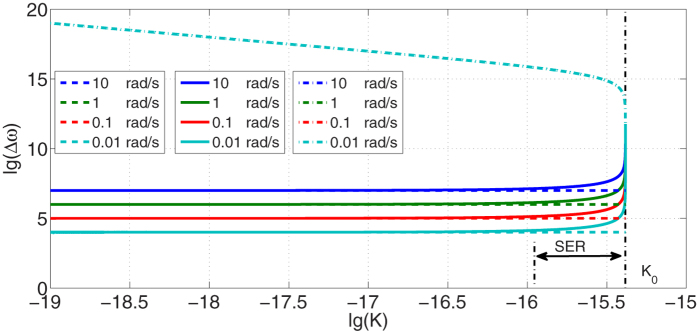
Results of 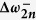 (dashed lines, bottom), 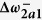 (solid curves, bottom) and 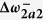 (dot dash curves, top) versus the dispersion gradient *K* in different angular velocities. Profiles of 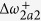 of different angular velocities overlap while their corresponding Δ*n* are beyond attainable value in experiments^24^. The SER refers to the strong enhancement region and *K*_0_ is the limitation for 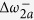.

The situation for 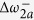 is slightly different, as *K* is confined by additional condition: 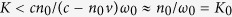. As is shown in Fig. 4, the situations of 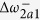 and 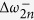 are similar to the those of 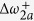 and 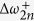, where evident amplification of RFS is achieved when *K* approaches *K*_0_. The profiles of 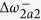 in different rotation velocities overlap, while the change of refractive index Δ*n* is beyond the available value in experiments^24^ too by the time, so only light with frequency 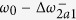 can exists in the CCW direction in practice.

**Figure 5:**
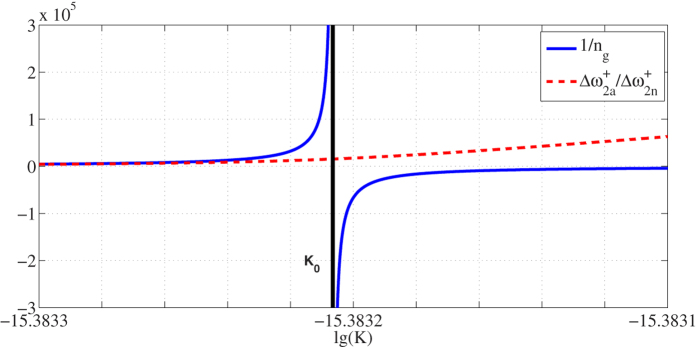
Results of 1/*n*_*g*_ (solid curves) and 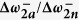 (dashed curves) versus the dispersion gradient *K* with angular velocity Ω = 10 rad/s.

The physical meaning of *K*_0_ may be understood that it is also the critical value of group index. If *K* > *K*_0_, group index will be negative according to its definition and the pulse envelope appears to travel backward^27^, which may result in the dramatic change of RFS. Similar conclusion was early demonstrated^19^, but we need to point out that RFS will not be “infinitely large” when using CAD (*n*_*g*_ = 0). Figure 5 clearly shows the difference between our results and the approximate amplification factor 1/*n*_*g*_. It can be seen that the approximation is inaccurate when K approaches the critical *K*_0_, which will result in overestimation of the amplification effect. Our rigorous expression of RFS avoids this defect and provides a precise theoretical guidance to achieve ultrasensitive ADEROGs. Moreover, there is no confusing “negative enhancement” in our results, which leads to clear and coherent explanations of the dispersion-enhanced mechanism.

As the signals of CW and CCW light should be detected simultaneously and the RFS is only effectively amplified in a tiny scope, where amplification factor rapidly changes as the variation of the dispersion gradient *K*, subtle control of the dispersion is hence of great importance when designing such ADEROGs. Furthermore, the properties of anomalous dispersion will influence not only the amplification of RFS, but also the detection range of such gyros. As we showed above, the critical *K*_0_ is approximately equal to *n*_0_/*ω*_0_ and thus can be manipulated by changing the original refractive index and resonant frequency. However, its magnitude is fixed at around 10^−15^ for traditional light sources and anomalous dispersion materials, which makes the SER be in the magnitude of 10^−15^ too. The attainable shift of refractive index Δ*n* in practice will therefore confine the width of RFS according to 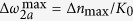. We can estimate the correspondingly maximum detectable angular velocity by combing the expression of *K*_0_ and Δ*ω*_2*n*_ while assuming the enhancement factor as 1/*n*_*g*_:


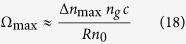


**Figure 6:**
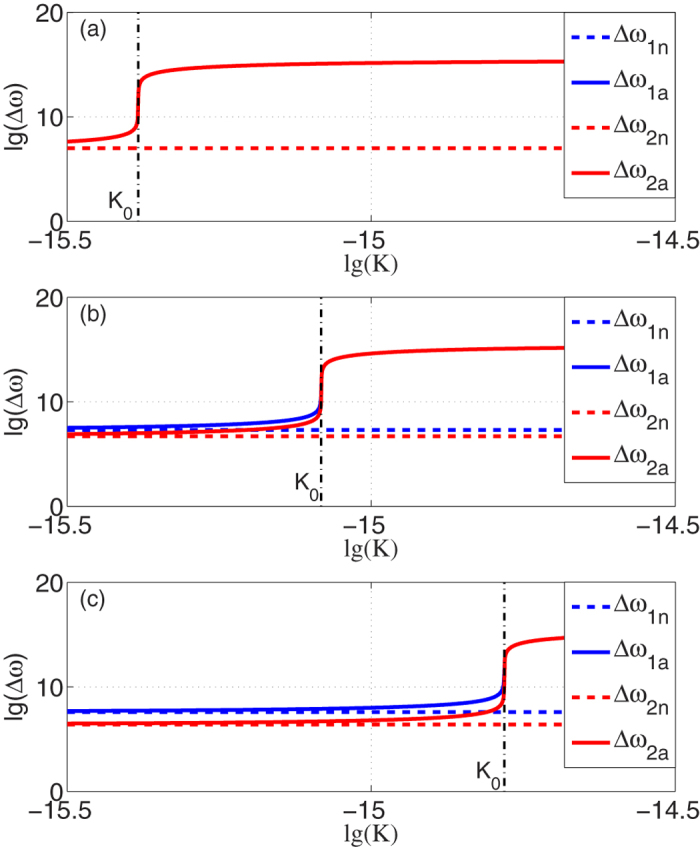
RFSs of 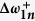 (blue dashed lines) and 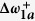 (blue solid curves), 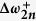 (red dashed lines), 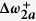 (red solid curves) versus the dispersion gradient *K* in different *n*_0_ with angular velocity Ω = 10 rad/s. *n*_0_ in (**a**–**c**) is 1, 2, 4 respectively. 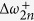 overlaps 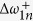 while 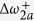 overlaps 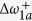 in (**a**) as modification factor is 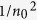.

We have discussed the results of relativistic RFSs above. The results and analyses for non-relativistic RFSs are similar to those for RFSs in relativistic cases except that a modification factor 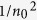 is introduced when considering STR, i.e. the RFS in non-relativistic cases is greater than those in non-relativistic cases. Figure 6 shows the results of 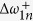, 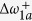, 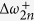 and 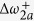 in different *n*_0_, while the perimeter of the resonator and original resonant wavelength are set as the prior cases. Obviously, the difference between 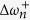 and 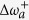 increases when the original refractive index *n*_0_ becomes larger. Although the influence of STR is negligible in the ADEROGs with atomic vapor as *n*_0_ ≈ 1, it matters in the gyroscopes’ design when other dispersive media are used.

## Methods

Theoretical analysis of the resonant frequency shift in anomalous dispersion enhanced resonant optical gyroscopes is carried out according to beam propagation theory. Numerical calculation is performed by MATLAB for showing the influence of parameters.

## Additional Information

**How to cite this article**: Lin, J. *et al*. Theoretical analyses of resonant frequency shift in anomalous dispersion enhanced resonant optical gyroscopes. *Sci. Rep.* **6**, 38759; doi: 10.1038/srep38759 (2016).

**Publisher's note:** Springer Nature remains neutral with regard to jurisdictional claims in published maps and institutional affiliations.
